# Deletion of the Neuronal Transcription Factor Satb1 Induced Disturbance of the Kinome and Mechanisms of Hypoxic Preconditioning

**DOI:** 10.3390/biology12091207

**Published:** 2023-09-04

**Authors:** Egor A. Turovsky, Viktor S. Tarabykin, Elena G. Varlamova

**Affiliations:** 1 Institute of Cell Biophysics of the Russian Academy of Sciences, Federal Research Center “Pushchino Scientific Center for Biological Research of the Russian Academy of Sciences”, 142290 Pushchino, Russia; 2 Institute of Neuroscience, Lobachevsky State University of Nizhny Novgorod, 23 Gagarin Ave., 603022 Nizhny Novgorod, Russia; victor.tarabykin@charite.de; 3 Institute of Cell Biology and Neurobiology, Charité—Universitätsmedizin Berlin, Charitéplatz 1, 10117 Berlin, Germany

**Keywords:** Satb1, calcium ions, protein kinases, gene deletion, neurons, cortex, signal transduction, epileptiform activity

## Abstract

**Simple Summary:**

In this work, we have shown for the first time that deletion of the transcription factor Satb1 in cortical neurons leads to changes in the expression of key protein kinases involved in the induction of epileptiform activity. As a result, neurons derived from mice with a Satb1 deletion are not only characterized by increased hyperexcitation but also by increased sensitivity to hypoxia and impaired hypoxic preconditioning mechanisms.

**Abstract:**

Genetic disorders affecting the functioning of the brain lead not only to the development of numerous hereditary diseases but also to the development of neurodegenerative and cognitive disorders. The result of this may be the disability of part of the able-bodied population. Almost all pathological states of the brain are characterized by serious defects in the intracellular and intercellular signaling of neurons and glial cells. At the same time, the mechanisms of disruption of these signaling cascades are not well understood due to the large number of molecules, including transcription factors that, when mutated, cause brain malformations. The transcription factor Satb1 is one of the least studied factors in the cerebral cortex, and the effects of its deletion in the postnatal brain are practically not studied. Hyperexcitability of neurons is observed in many genetic diseases of the nervous system (Hirschsprung’s disease, Martin–Bell syndrome, Huntington’s disease, Alzheimer’s, etc.), as well as in ischemic brain phenomena and convulsive and epileptic conditions of the brain. In turn, all these disorders of brain physiology are associated with defects in intracellular and intercellular signaling and are often the result of genetic disorders. Using Satb1 mutant mice and calcium neuroimaging, we show that Satb1 deletion in projection neurons of the neocortex causes downregulation of protein kinases PKC, CaMKII, and AKT/PKB, while a partial deletion does not cause a dramatic disruption of kinome and Ca^2+^ signaling. As a result, Satb1-null neurons are characterized by increased spontaneous Ca^2+^ activity and hyperexcitability when modeling epileptiform activity. As a result of the deletion of Satb1, preconditioning mechanisms are disrupted in neurons during episodes of hypoxia. This occurs against the background of increased sensitivity of neurons to a decrease in the partial pressure of oxygen, which may indicate the vulnerability of neuronal networks and be accompanied by impaired expression of the Satb1 transcription factor. Here, we show that Satb1 deletion impaired the expression of a number of key kinases and neuronal hyperexcitation in models of epileptiform activity and hypoxia.

## 1. Introduction

The cerebral cortex is the crown of mammalian evolution and is the center of higher cognitive abilities that distinguish humans from other animal species [1]. The development of the cerebral cortex is a complex and highly organized process. Disruption of any step in this process can lead to a range of neurodevelopmental disorders. Many of these disorders are caused by malformations of the cerebral cortex [2]. Malformations of the cerebral cortex often cause epilepsy, developmental delay, neurological deficits, and mental retardation in humans. In the last two decades, significant progress has been made in identifying the genes that control various aspects of the development of the cerebral cortex [3]. At the same time, the functions of many transcription factors are not well understood.

Transcription factor Satb1 (a special AT-rich sequence-binding protein) is involved in the organization of chromatin and the regulation of the differentiation of stem cells, neurons, and osteoblasts. In the cerebral cortex, it is known to regulate the migration and maturation of somatostatin-positive interneurons, synaptogenesis, and the differentiation of neurons [4]. Satb1 is widely expressed in the neocortex, nucleus of the diagonal bundle, amygdala, hippocampus, and spinal cord. At the same time, Satb1-positive neurons are found in very small numbers in the substantia nigra of the midbrain and predominantly in dopaminergic neurons. Different levels of Satb1 expression were also detected in different subtypes of neurons. For example, a high level of Satb1 expression was found in somatostatin-positive, calretinin-positive, and neuropeptide Y-positive interneurons, while no Satb1 expression was detected in vasoactive intestinal peptide-positive interneurons [4,5]. Satb1 regulates the expression of a number of genes in the cerebral cortex—ERRB2, ABL1, MMP2, E-CADHERIN, VEGFB, TGFB1, and KISS1 [6]—as well as genes encoding subunits of excitatory glutamate receptors [7].

Mice with the Satb1 mutation are characterized by incomplete eye opening and an increased grasping reflex [8]. In mice with a complete deletion of Satb1, the development of the cerebral cortex occurs without significant disturbances, but for pyramidal neurons in the postnatal period, a decrease in the density of dendritic spines has been shown. In addition, the Satb1 transcription factor regulates a number of early development genes, Fos, Fosb, Egr1, Egr2, Arc, Bdnf, etc., which are also involved in synaptic plasticity [9].

The aim of this study was to investigate the role of the transcription factor Satb1 in the regulation of expression of key protein kinases, hyperexcitation of neurons in the cerebral cortex, and activation of the phenomenon of hypoxic preconditioning.

## 2. Materials and Methods

The study was performed in accordance with the permission of the Commission on Bioethics of the Institute of Cell Biophysics of the Russian Academy of Sciences (Act708n from 23 August 2010). During the course of the study, the Acts of Humane Treatment of Laboratory Animals were observed (Council Directive 2010/63 EU).

### 2.1. Animals

The generation of Satb1 knockout mice is described in detail by Close et al. and Goebbels et al. [10,11]. For experiments, Satb1 knockout mice were kindly provided from the SPF animal facility at the Lobachevsky State University of Nizhni Novgorod and from the Institute of Cell Biology and Neurobiology, Charité—Universitätsmedizin Berlin. Newborn mice were used to obtain cell cultures. The tails of mice were kept at −80 °C for subsequent genotyping. Isolation of DNA from tails and genotyping by PCR were carried out in accordance with the methods described by us earlier [7]. The following primers were used to determine the amplified product. Satb1-floxed allele and wild-type allele: 5′-GCATGTTTGTCTGTGTGCC-3′; 5′-CAGAAACAGTCTGGAGGGAGG-3′. Amplification program was as follows: 95 °C, 3 min; 95 °C, 30 s; 55 °C, 30 s; 72 °C, 30 s; 72 °C, 5 min; 30 cycles. The wild-type allele product is ∼120 bp, knockout ∼160 bp. Primers for amplification of the NexCre allele and the wild-type allele: 5′-CCG CATAACCAGTGAAACAG-3′; 5′-GAGTCCTGGAATCAGTCTTTTC-3′; 5′-AGAATGTGGAGTAGGGTGAC-3′. Amplification program: 95 °C, 3 min; 95 °C, 20 s; 54 °C, 30 s; 72 °C, 60 s; 72 °C, 2 min; 35 cycles. The product of the wild-type allele is ∼750 bp, the NexCre allele is ∼500 bp.

### 2.2. Cell Culture Preparation

The method for obtaining neuroglial cell cultures from the mice’s cerebral cortex was described in detail by us earlier [12]. Briefly, the cerebral cortex of neonatal (male) mice was removed and minced in a test tube and incubated for 15 min at 37 °C in a solution consisting of 0.1% trypsin in Versene without Ca^2+^ and Mg^2+^ ions. Trypsin activity was inactivated by adding fetal calf serum, and the cell suspension was centrifuged at 300× *g* for 3 min. The supernatant was removed, and the cells were resuspended in a culture medium consisting of Neurobasal-A medium (Thermo Fisher Scientific, Waltham, MA, USA), 0.5 mM glutamine (Sigma-Aldrich, Burlington, MA, USA), 2% B-27 supplement (Thermo Fisher Scientific), and 20 μg/mL gentamicin (Sigma-Aldrich). The procedure was repeated twice. After that, 200 μL of the cell suspension was placed on round cover slips with a diameter of 25 mm (VWR International, Radnor, PA, USA) coated with polylysine and placed in Petri dishes. Five hours after the cells were attached to the glass, 1.5 mL of the culture medium was added to Petri dishes. Every 3 days, 2/3 of the culture medium was replaced with fresh medium. Cells were cultured for up to 10–14 days in vitro (DIV).

### 2.3. Fluorescent Ca^2+^ Measurements

To measure [Ca^2+^]_i_ dynamics, cortical cells were loaded with a Fura-2AM fluorescent probe (Thermo Fisher Scientific), and [Ca^2+^]_i_ changes were recorded using an inverted motorized Axiovert 200M fluorescent microscope (Carl Zeiss, Oberkochen, Germany) [12]. Cortical neurons were loaded with Fura-2 (5 μM) for 40 min at 37 °C in Hank’s medium (in mM: 156 NaCl, 3 KCl, 2MgSO_4_, 1.25 KH_2_PO_4_, 2CaCl_2_, 10 glucose) balanced with 10 mM HEPES. After that, the cells were washed from the probe for 15 min. Fura-2 fluorescence was excited and recorded using excitation filters BP340/30 and BP387/15, beam splitter FT-409 and emission filter BP510/90, objective lens Plan-Neo fluar 10×/0.3, and excitation light source HBO 103W/2. Fluorescence images were acquired at 1 frame per second using a high-speed monochrome CCD camera AxioCam HSm with a high-speed light filter replacing system, Ludl MAC5000. The Ca^2+^ signals of neurons are presented as the ratio of the Fura-2 fluorescence intensity in the 340 nm recording channel to the 380 nm recording channel. Image series were processed and analyzed using the ImageJ 2002 software package.

### 2.4. The Technique for Short-Term Hypoxia Episode Generation

The experimental model of neuronal preconditioning using repeated episodes of hypoxia/reoxygenation was described in detail earlier [13]. To induce hypoxia, oxygen was displaced from the Hank’s medium in a special hermetic system by bobbling with argon. During episodes of hypoxia, a hypoxic medium was supplied to the experimental chamber with cells of the cerebral cortex with a constant supply of argon to avoid contact of the medium with atmospheric oxygen. Reoxygenation was carried out by supplying oxygen-saturated medium to the cells. For experiments on modeling episodes of hypoxia/reoxygenation, hypoxia was applied for 3 min, and reoxygenation periods were 10 min. Such episodes of hypoxia/reoxygenation were performed three times. The effect of episodes of hypoxia/reoxygenation on neurons and the formation of the phenomenon of hypoxic preconditioning were assessed by the change in the amplitudes of the Ca^2+^ signals of neurons on the activation of NMDA receptors, created by a short-term (30 s) application of 10 μM NMDA in a magnesium-free medium before hypoxia/reoxygenation and after each of the three episodes of hypoxia/reoxygenation.

### 2.5. Extraction of RNA and Real-Time Polymerase Chain Reaction (RT-qPCR)

Isolation of total RNA from cortical cultures was performed using the MagMAX mirVana kit (Thermo Fisher Scientific). The quality of the obtained RNA was determined using electrophoresis, and the concentration of RNA was determined on a NanoDrop 1000c spectrophotometer. Reverse transcription was performed using the RevertAid H Minus First Strand kit (Thermo Fisher Scientific). Amplification was performed using the DTlite Real-Time PCR System (DNA-technology, Moscow, Russia) in a 25 µL mixture containing 5 µL of qPCRmix-HS SYBR (Evrogen, Moscow, Russia, Cat. #PK147L), 1 µL (0.2 µM) of the primer solution, 18 µL water (RNase-free), 1 µL cDNA. Amplification process consolidated the initial 5 min denaturation at 95 °C, 40 cycles of 30 s denaturation at 95 °C, 20 s annealing at 60–62 °C, and 20 s extension steps at 72 °C. The final extension was performed for 10 min at 72 °C [12]. All the sequences were designed based on the analysis of the nucleotide sequences of the existing gene isoforms and are specific for the mouse with FAST PCR 5.4 and NCBI Primer-BLAST software (https://www.ncbi.nlm.nih.gov/tools/primer-blast/primertool.cgi, accessed on 6 July 2023). The data were analyzed with DTlite software (https://dna-technology.com/sites/default/files/dtprime_dtlite_v06_part_2.pdf, accessed on 6 July 2023; DNA-technology, Moscow, Russia) and Origin 8.5 software (OriginLab Corporation, Northampton, MA, USA). The expression of the studied genes was normalized to gene encoding Glyceraldehyde 3-phosphate dehydrogenase (GAPDH) and was presented in relation to control mice. Data were analyzed using Livak’s method.

### 2.6. Immunocytochemistry

The immunocytochemical method was used to detect PI3K and BDNF in neurons [12]. Cells were fixed for 20 min in 4% paraformaldehyde with 0.25% glutaraldehyde, then washed three times for 5 min in ice-cold PBS. Cells were permeabilized in 0.1% Triton X-100 solution for 15 min. Next, the cells were incubated for 30 min with 10% donkey serum at room temperature. Primary antibodies were added for 12 h at 4 °C. After loading with primary antibodies, cells were washed in PBS three times for 5 min and secondary antibodies conjugated with a fluorescent label were added. We used mouse anti-NeuN (1:200; Abcam, Cambridge, UK) antibodies to identify neurons, chicken anti-BDNF antibodies (1:150; Abcam, Cambridge, UK) and eliminated rabbit monoclonal antibodies to PI3-Kinase p85 alpha (1:200; Abcam, UK) were used to determine the level of BDNF and PI3K in neurons. We used donkey polyclonal secondary antibody to rabbit IgG (H + L) (Alexa Fluor-647) (Jackson ImmunoResearch Europe LTD, Cambridge, UK, RRID: AB_2492288), donkey anti-chicken Alexa Fluor-488 conjugated antibodies (Abcam, UK), and donkey polyclonal secondary antibody to mouse IgG-H&L (Alexa Fluor-594) (Abcam, RRID: AB_2732073). The fluorescence of secondary antibodies was recorded using an inverted confocal microscope Leica TCS SP5 (Leica, Wetzlar, Germany).

### 2.7. Statistical Analysis

All presented data were obtained from at least three cell cultures from 2–3 different passages. All values are given as mean ± standard error (SEM). Statistical analyses were performed by paired *t*-test. MS Excel (Microsoft Office 2016, Redmond, Washington, USA), ImageJ (https://imagej.nih.gov/ij/download.html (accessed on 18 May 2023), Java 1.6.0_12, RRID: SCR_003070, LOCI, University of Wisconsin, Madison, WI, USA), Origin 2016 (OriginLab, Northampton, MA, USA), and Prism GraphPad 7 (GraphPad Software, RRID: SCR_002798) software was used for data and statistical analysis.

## 3. Results

### 3.1. Deletion of the Transcription Factor Satb1 in Cortical Neurons Leads to Impaired Expression of Key Protein Kinases and Genes Regulating Cell Viability

**Figure 1:**
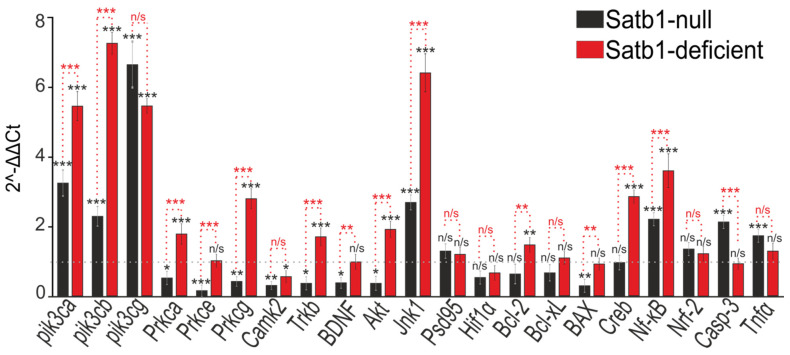
Expression level of mRNA of genes encoding protein kinases, transcription factors, and apoptosis regulatory proteins in brain cortex cells obtained from Satb1-null (null) and Satb1-deficient (deficient) mice. The levels of mRNA expression in cortical cells of control mice (WT) are taken as 1 and are represented as a dotted line. mRNA was obtained from primary cell cultures of the cerebral cortex of mice after 10 days of cultivation. Statistical significance was assessed using t-test. Comparison of experimental groups vs. control: n/s—data not significant (*p* > 0.05), * *p* < 0.05, ** *p* < 0.01, and *** *p* < 0.001, n = 3.

Cortical cells were grown up to day 10 in vitro (DIV), after which total RNA was isolated from the cultures and PCR analysis was performed. An increase in the expression of genes encoding isoforms of various proteins was detected in the cortical cells isolated from Satb1-deficient mice as compared with cells from the wild-type mice. Protein kinases PI3K (pik3ca, pik3cb, pik3cg) were increased by 5–7-fold, PKC (Prkca, Prkcg) by 2–3.5-fold, Akt by 2-fold, and Jnk1 by 6.5-fold. At the same time, expression of the genes encoding CaMKII (Camk2) decreased in Satb1-deficient cells (Figure 1, red bars). Whereas cells isolated from Satb1-null mice (complete deletion of Satb1) had a decrease in the expression of genes encoding PKC and a more pronounced decrease in CaMKII and Akt. The expression of PI3K and Jnk1 was higher as compared to the wild type (Figure 1, black bars). The expression of genes encoding some transcription factors and apoptosis regulatory proteins was also affected by the deletion of Satb1. In Satb1-deficient cells, the expression of Trkb, Bcl-2, Creb, and Nf-kB mRNA increased (Figure 1, red bars). Whereas in Satb1-null cells, a decrease in the expression of Trkb and BAX mRNAs and an increase in the expression levels of the Nf-kB, Casp-3, and Tnfa genes were observed (Figure 1, black bars).

**Figure 2:**
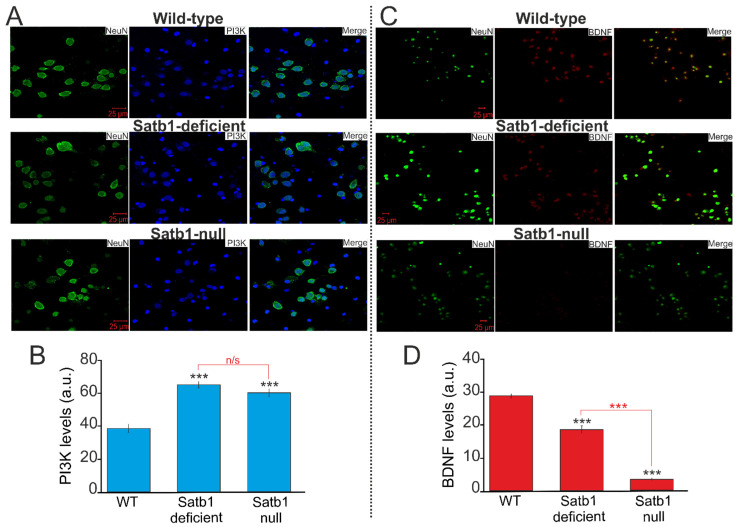
Immunocytochemical staining of cortical neurons derived from wild-type (WT), Satb1-deficient, and Satb1-null mice with antibodies against phosphoinositide 3-kinase (PI3K) (**A**) and brain-derived neurotrophic factor (BDNF) (**C**). NeuN is a neuronal marker. (**B**,**D**) Intensity levels of PI3K (**B**) and BDNF (**D**) were determined by confocal imaging. We analyzed individual cells that had fluorescence of secondary antibodies. The quantitative data reflecting the level of PI3K or BDNF expression are presented as fluorescence intensity values in summary bar charts (mean +/− SEM). The values were averaged by 150 cells for each column. The results obtained after immunostaining agree well with the data of fluorescence presented in panel (**A**,**C**). Statistical significance was assessed using paired *t*-test. *** *p*-level < 0.001, n/s—data not significant (*p* > 0.05).

The results of immunocytochemical staining of cell cultures with antibodies against phosphoinositide 3-kinase (PI3K) (Figure 2A) and BDNF (Figure 2C) showed that the content of the PI3K protein in neurons derived from Satb1-deficient and Satb1-null mice is higher compared to neurons in WT mice (Figure 2B). At the same time, the content of BDNF in Satb1-deficient and Satb1-null neurons decreased compared to WT neurons (Figure 2D). Moreover, BDNF expression in Satb1-null neurons is critically low, both in comparison with WT neurons and in comparison, with Satb1-deficient neurons.

Thus, complete and partial deletion of the transcription factor Satb1 had different effects on the expression of protein kinases and genes regulating cell viability. A deficiency of Satb1, in general, led to a compensatory increase in the expression of most of the studied protein kinases. Complete deletion of Satb1, in general, led to some increase in the expression of most of the studied protein kinases. Complete deletion of Satb1, on the contrary, caused a decrease in their expression and a trend toward increased expression of genes encoding proapoptotic proteins.

### 3.2. Deletion of the Transcription Factor Satb1 Correlates with Hyperexcitation of Cortical Neurons

**Figure 3:**
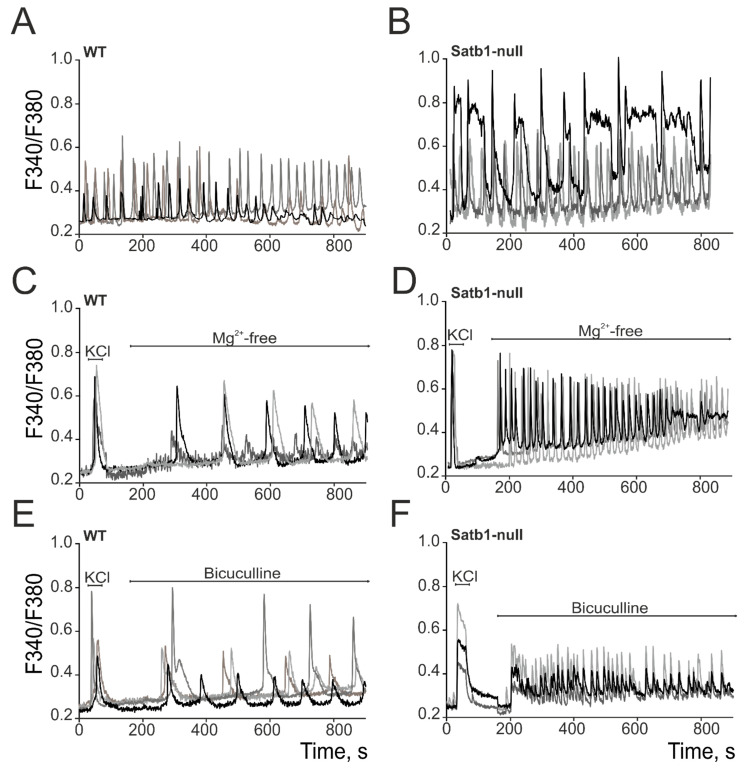
Generation of spontaneous (**A**,**B**), Mg^2+^-free-induced (**C**,**D**), and bicuculline-induced (**E**,**F**) Ca^2+^ oscillations in cortical neurons obtained from control (WT) and Satb1-null mice. Typical Ca^2+^ signals of neurons (different colors) in one experiment are presented. At the beginning of the experiment, a short-term application of 35 mM KCl was performed to identify neurons in culture.

The effects of the Satb1 deletion on the expression of the studied genes were most dramatic in Satb1-null neurons; therefore, it was decided to carry out further experiments on cultures obtained from the cerebral cortex of Satb1-null mice. Analysis of the spontaneous Ca^2+^ activity of cortical neurons from wild-type (WT) mice and Satb1-null neurons showed that spontaneous Ca^2+^ activity was recorded in WT and Satb1-null neurons on the 14th day of cultivation. The frequency of spontaneous Ca^2+^ oscillations in both experimental groups did not differ significantly, but the amplitude of Ca^2+^ pulses was higher in Satb1-null neurons (Figure 3B) compared to WT neurons (Figure 3A).

On day 10 of the cultivation, the generation of spontaneous Ca^2+^ activity was not yet registered in neurons (Figure 3C–F). Modeling of neuronal epileptiform activity by replacing the medium with magnesium-free medium or adding 10 µM bicuculline showed that Ca^2+^ oscillations were generated in WT (Figure 3C,E) and Satb1-null neurons (Figure 3D,F). At the same time, in the magnesium-free model of epileptiform activity, the amplitude and frequency of Ca^2+^ oscillations were lower in WT neurons (Figure 3C) compared to Satb1-null neurons (Figure 3D). When bicuculline was added to Satb1-null neurons, high-frequency Ca^2+^ oscillations occurred with a high degree of synchronization (Figure 3F), while in WT neurons, Ca^2+^ oscillations were asynchronous and low-frequency, but with high amplitudes (Figure 3E).

Thus, neurons derived from the cerebral cortex of mice with a complete deletion of Satb1 are characterized by increased spontaneous Ca^2+^ activity and are also characterized by a higher frequency of Ca^2+^ oscillations when modeling epileptiform activity.

### 3.3. Deletion of the Transcription Factor Satb1 Affects the Sensitivity of Cortical Neurons to Hypoxia and the Induction of Hypoxic Preconditioning

**Figure 4:**
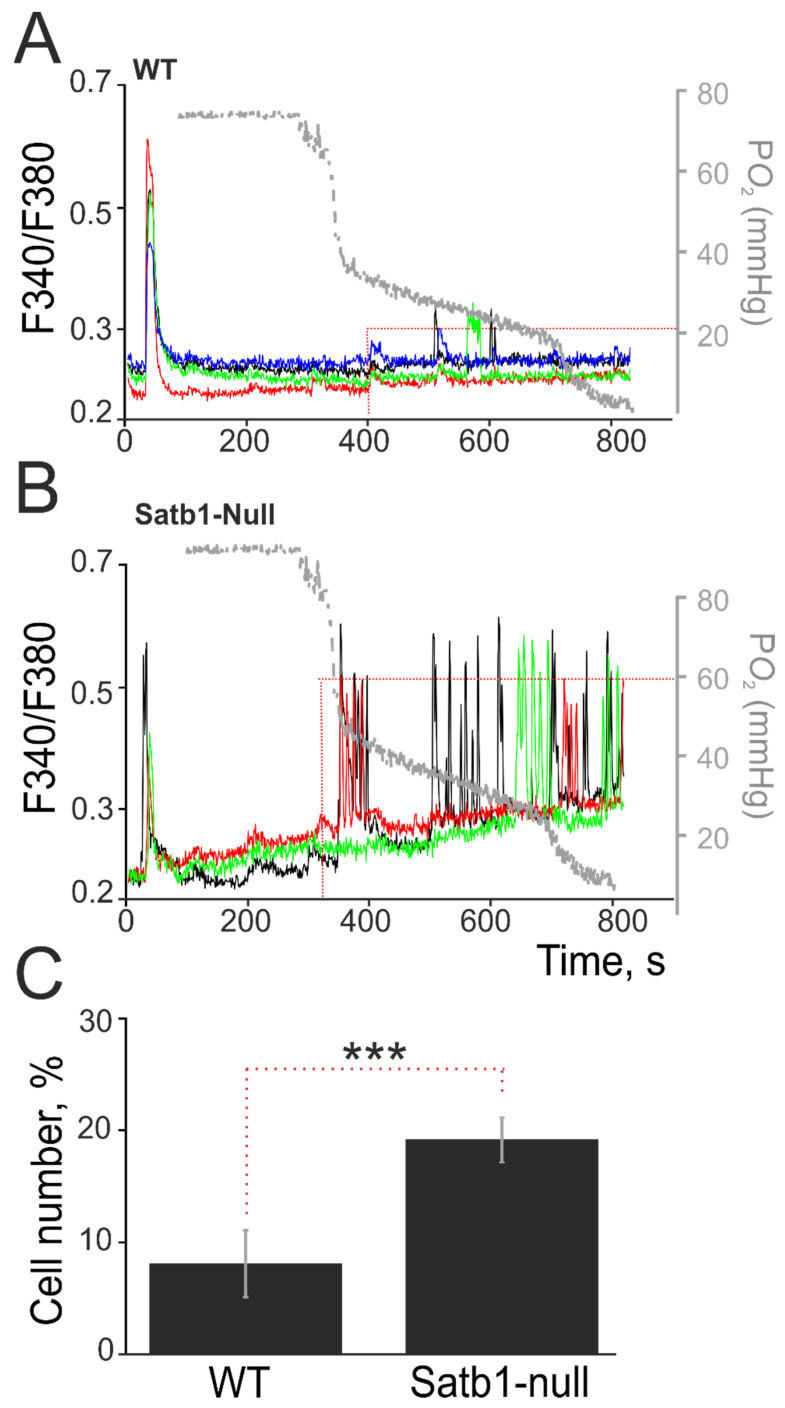
Effect of hypoxia on control (WT) and Satb1-null neurons of the cerebral cortex. A decrease in the partial pressure of oxygen (_P_O_2_) in the extracellular environment causes an increase in [Ca^2+^]_i_ in cultured cortical neurons derived from wild-type mice (**A**, WT) and Satb1-null mice (**B**). (**C**) The number of neurons (expressed as a percentage of the total number in the microscope field of view) that respond with an increase in [Ca^2+^]_i_ during hypoxia simulation. To identify neurons, short-term depolarization was performed with 35 mM KCl. Typical Ca^2+^ signals of neurons (different colors) in one experiment are presented. For clarity, the Ca^2+^ responses of neurons are plotted on the same scales along the y-axes. The gray y-axes and the curve on the graph (on the right) show the decrease in oxygen partial pressure (_P_O_2_, mmHg) in the cells during the experiment, measured using a somatic oximeter. Statistical significance was assessed using *t*-test. *** *p* < 0.001. N (cell cultures) = 3. n (repetitions) = 6.

Neurons were distinguished from astrocytes using a depolarizing stimulus with the short-term application of 35 mM KCl, to which only neurons responded with an increase in [Ca^2+^]_i_. Further, using the self-made vacuum system, hypoxia conditions were created by displacing oxygen dissolved in the experimental chamber. Simultaneously with the registration of [Ca^2+^]_i_, the partial pressure of oxygen (_P_O_2_) was measured using a somatic oximeter. The threshold value for the decrease in _P_O_2_ was considered to be the beginning of the generation of Ca^2+^ responses in neurons. The appearance of the first Ca^2+^ responses to hypoxia in WT neurons occurred in 8–10% of cells (Figure 4A,C) when the partial pressure of oxygen decreased to 40 mmHg, while the rest of the cells in the microscopic field of view did not react. Satb1-null neurons began to respond to hypoxia at 60 mmHg, and the Ca^2+^ responses were in the form of high-amplitude Ca^2+^ oscillations (Figure 4C) and were recorded in more than 15% of the cells (Figure 4C). At the same time, Satb1-null neurons reacted to the appearance of high-frequency Ca^2+^ oscillations with the release of the base level [Ca^2+^]_i_ to a new stationary level already at a decrease in _P_O_2_ to 75–80 mmHg. Taking into account the high percentage (more than 20%) of responding neurons, one can speak of an increased sensitivity of Satb1-null neurons to hypoxia.

Short-term episodes of hypoxia protect neurons from death during subsequent global hypoxia and ischemia, reducing the high concentration of [Ca^2+^]_i_ during this period [13] through the activation of hypoxic preconditioning mechanisms. Hypoxic preconditioning is an effective way to increase the resistance of organs and tissues to the effects of prolonged hypoxia–reoxygenation and ischemia using one [14] or several episodes of short-term hypoxia followed by reoxygenation [15].

**Figure 5:**
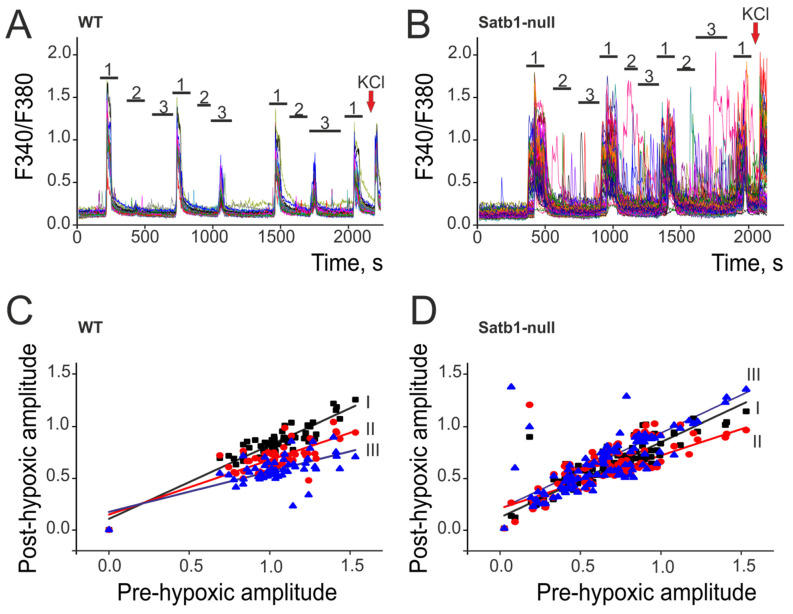
Induction of the hypoxic preconditioning phenomenon in cerebral cortex neurons derived from control (WT) and Satb1-null mice. (**A**,**B**) Ca^2+^ signals of cerebral cortex neurons obtained from control (**A**, WT) and Satb1-null (**B**) mice after application of 10 μM NMDA in a magnesium-free medium (1), episodes of short-term hypoxia (2), and periods of reoxygenation (3). (**C**,**D**) Dependence of the amplitude of Ca^2+^ signals of WT neurons (**C**) and Satb1-null neurons of the cerebral cortex on application of NMDA in a magnesium-free medium after episodes of short-term hypoxia (*y*-axes) on the pre-hypoxic amplitude of Ca^2+^ signals (*x*-axes). The amplitudes of Ca^2+^ responses and their approximations by a linear function after each of the three episodes of short-term hypoxia are presented. I, II, III: Approximation of neuronal Ca^2+^ signal amplitudes to NMDA application by a linear function after each of the three episodes of hypoxia. Equation for linear function: y = a + b*x. For clarity, the figures are presented in the same *x*- and *y*-axes scales. For panels (**C**,**D**), black squares are the amplitudes of the Ca^2+^ signals of individual neurons after the first episode of hypoxia/reoxygenation; red circles: amplitudes of Ca^2+^ signals of neurons after two episodes of hypoxia/reoxygenation; blue triangles: amplitudes of Ca^2+^ signals of neurons after three episodes of hypoxia/reoxygenation.

Using fluorescence microscopy in cortical neurons stained with the calcium-sensitive probe Fura-2, we recorded changes in [Ca^2+^]_i_ upon activation of excitatory ionotropic NMDA glutamate receptors (NMDAR) before and after episodes of hypoxia–reoxygenation. Activation of NMDAR by the short-term (30 s followed by 10-fold longer periods of washing) application of a selective agonist, N-methyl-D-aspartate (NMDA, 10 μM, Figure 5), in a magnesium-free medium caused the appearance of Ca^2+^ responses of various amplitudes in each individual neuron. Previously, we have shown that repeated episodes of activation of these receptors always cause Ca^2+^ responses that are repeated in form and amplitude in individual neurons. This is a characteristic of the activity of NMDA receptors, while the slope of the linear function approximating these Ca^2+^ responses is close to 1 [13]. Therefore, changes in the amplitudes of Ca^2+^ responses and the tangent of the slope of the linear function will make it possible to reveal the effects of hypoxia–reoxygenation on the neuronal network.

To reveal the effects of hypoxia on cultured WT (Figure 5A) and Satb1-null neurons (Figure 5C), the first (pre-hypoxic) activation of NMDAR (NMDA at a concentration of 10 μM) was performed in a magnesium-free medium (designation 1), after which an episode of hypoxia (mark 2) was performed for 3 min, followed by a 10 min reoxygenation period (mark 3). During the experiment, the cells were subjected to episodes of hypoxia and reoxygenation three times, and after each reoxygenation, NMDAR was activated (post-hypoxic Ca^2+^ responses). It is interesting that WT neurons did not respond with Ca^2+^ signals to hypoxia and reoxygenation (Figure 5A), while powerful Ca^2+^ signals were recorded in Satb1-null neurons during these periods, and the amplitudes of these signals increased with each subsequent episode of hypoxia/reoxygenation (Figure 5B).

After episodes of hypoxia–reoxygenation in WT neurons, in response to the application of NMDA, Ca^2+^ signals consistently decreased, which is confirmed by a decrease in the slope of the straight line approximating the amplitude of Ca^2+^ signals (Figure 5C): 0.7 ± 0.05 (after the first episode of hypoxia), 0.52 ± 0.05 (after the second episode of hypoxia), and 0.38 ± 0.06 (after the third episode of hypoxia). In Satb1-null neurons, Ca^2+^ signals to NMDA application after episodes of hypoxia–reoxygenation decreased more moderately, and after episode three, the amplitudes of Ca^2+^ signals increased, as evidenced by the slope coefficients of the linear approximation of the amplitudes (Figure 5D): 0.87 ± 0.05 (after the first episode of hypoxia), 0.78 ± 0.06 (after the second episode of hypoxia), and 1.19 ± 0.06 (after the third episode of hypoxia).

Thus, impaired expression of the transcription factor Satb1 correlates with an increased sensitivity of neurons to a decrease in oxygen partial pressure and a disruption in the mechanisms of hypoxic preconditioning, which is determined by the expression of protein kinases.

## 4. Discussion

Spontaneous Ca^2+^ activity of neurons during CNS development influences such developmental processes as cell migration, synaptogenesis, and neuronal maturation [16]. It was found that Satb1 expression was weakly expressed 24 h after seeding in GABAergic neurons isolated from the hindbrain of mouse embryos, but the percentage of Satb1^+^-GABAergic neurons increased during cultivation. At the same time, a depolarizing stimulus with the application of 40 mM KCl led to an increase in [Ca^2+^]_i_ in neurons through voltage-dependent L-type Ca^2+^ channels [17] and was accompanied by a significant increase in the level of Satb1 expression.

In our case, the deletion of Satb1 in cortical glutamatergic neurons led to more pronounced spontaneous Ca^2+^ oscillations as well as a greater “tendency” of the neural network to induce epileptiform activity. This can also be confirmed by data on the selective deletion of Satb1 in inhibitory interneurons, which led to damage to the mechanisms of inhibition of pyramidal neurons. At the in vivo level, this manifested itself in the induction of epileptiform activity in the cortical layers of the brain [18]. Interestingly, in cortical slices obtained from mice with conditional knockout, in which the transcription factor Satb1 is selectively deleted in SST-expressing interneurons, the authors were able to show increased spontaneous activity of neurons. In addition, slices from mutant animals manifested shorter latency for the expression of stable seizure-like events when using the model of epileptiform activity with low magnesium [19].

It is also known that the complete deletion of Satb1 in SST^+^ interneurons led to an abnormal integration of these interneurons during network development, which caused their damage and death [10]. Similarly, in our experiments, the complete deletion of Satb1 correlated with a decrease in the expression of genes encoding key protein kinases responsible for cell survival and coincided with an increase in the expression of pro-inflammatory genes. As for sensitivity to hypoxia, populations of GABAergic neurons that are most sensitive to hypoxia are known [20]. Since, as mentioned above, the expression of Satb1 is reduced in GABAergic neurons, the complete deletion of Satb1 in all neurons of the cerebral cortex, in our case, can change the phenotype of glutamatergic neurons and increase their sensitivity to hypoxia.

Hypoxic preconditioning is the short-term and repeated creation of hypoxic conditions that increase resistance to lethal hypoxia or global ischemia in various organs [21]. For such adaptation to long-term hypoxia, there are no pre-active mechanisms in the body; there are only genetically determined prerequisites that are implemented under certain conditions. Under oxygen deficiency, various protective mechanisms are activated in the brain cells, which make it possible to avoid irreversible disturbances in the functioning of tissues and organs. These include an increase in the power of stress-limiting systems and systems of oxygen transport and ATP resynthesis, an acceleration of the transformation of short-term memory into a long-term one, an increase in the synthesis of RNA and protein in brain cells, activation of the cytochrome p-450 detoxification system, activation of the antioxidant system, a decrease in the content of immune complexes in the blood, antiallergic and anti-blastoma effects, etc. [22]. Deletion of Satb1 leads to inactivation of the phenomenon of hypoxic preconditioning, in which neurons of the cerebral cortex, firstly, are characterized by increased Ca^2+^ activity under conditions of hypoxia/reoxygenation and, secondly, episodes of hypoxia do not suppress the amplitude of the Ca^2+^ response to the application of NMDA.

Partial deletion of Satb1 leads to a strong increase in the expression of the Prkcg gene, which encodes PKCγ. This isotype of PKC is most common in the nervous tissue and is involved in neuroprotection by regulating the expression of a number of glutamate receptor subunits, such as Gria4/GluR4 and Grin1/NMDAR1 [23]. The decrease in the expression of the α and ε isotype of PKC observed in this case may be a consequence of the insufficient maturity of the neuronal network, since a high level of PKCε expression is indicated for the final stages of neuronal development [24]; however, an increased level of PKCγ can play, in our case, a compensatory role. At the same time, the complete deletion of Satb1 leads to a decrease in the expression of genes encoding all PKC isotypes, which undoubtedly contributes to impaired neurotransmission and hyperexcitation. Increased JNK expression upon deletion of Satb1 may, under certain conditions, promote apoptosis. JNK has been shown to be involved in NMDAR-dependent neuronal apoptosis after stroke [25]. Mice lacking JNK3, an isoform of JNK that is highly expressed in the brain, are resistant to excitotoxic neuronal apoptosis, and the peptide inhibitor Tat-JBD 20 (JNK inhibitor-1) significantly suppresses apoptosis in various pathologies [26,27]. At the same time, we observe trends towards increased expression of genes encoding proapoptotic proteins caspase-3 and Tnfα.

One of the key stages in the induction of hypoxic preconditioning is the signaling pathway, during which information is transmitted from the oxygen level sensor to effectors through secondary messengers and a cascade of intracellular signaling proteins (phospholipases, protein kinases, transcription factors) [28]. Hypoxic preconditioning is accompanied by the phosphorylation of protein kinase C (PKC) and its translocation to the membrane [29]. In addition, the expression of PKCε and PKCβ increases after hypoxic preconditioning [30]. In this case, the complete deletion of Satb1 leads to the suppression of PKC expression, which correlates with impaired hypoxic preconditioning of neurons. Also, after hypoxic preconditioning, there is an increase in Akt kinase phosphorylation, which is suppressed by PI3K inhibitors and leads to the abolition of preconditioning [31,32]. The expression of this kinase is also suppressed by the deletion of Satb1.

The increased expression of phosphoinositide 3-kinase (PI3K) subunits observed with complete and partial deletion of Satb1 may create conditions for the functioning of mutant neurons against the background of increased sensitivity to NMDAR agonists since PI3K/Akt inhibits pro-death genes under conditions of excessive entry of Ca^2+^ ions through NMDAR [33]. Moreover, the PI3K signaling pathway plays an important role in maintaining Ca^2+^ homeostasis, regulating NMDA-dependent neuroplasticity, and suppressing apoptosis [20,34,35]. The most important and studied in terms of involvement in the activation of the preconditioning phenomenon is HIF-1α [28]. Along with mechanisms regulated by the oxygen level, HIF-1α expression can also occur through oxygen-independent mechanisms involving MAPK (mitogen-activated protein kinase) and PI3K kinases [36]. Of particular importance is PI3K, which belongs to the group of RISK kinases (protecting against reperfusion damage) and is involved in the inhibition of the mitochondrial pores’ opening [37]. However, deletion of Satb1 does not affect HIF-1α expression, and an increase in PI3K expression does not appear to be sufficient to induce hypoxic preconditioning.

Decreased expression of CaMKII, a kinase associated with NMDAR regulation, may contribute to neural network hyperexcitation upon deletion of Satb1. It has been established that CaMKII is activated (at T286 on CaMKIIα subunits or T287 on CaMKIIβ subunits) in response to the entry of Ca^2+^ ions through NMDAR [38], and the T286A mutation that prevents the autophosphorylation and activation of CaMKIIα in mice significantly impairs NMDAR-dependent LTP in the hippocampal CA1 area and memory performance in a Morris water maze task [39]. Moreover, it has been shown that overexpressing a GluN2B carboxyl-terminal fragment (839–1482aa) that disrupts the physiological interaction of NMDAR/CaMKII leads to severe deficits in hippocampal LTP and spatial learning in transgenic mice [40]. Deletion of the transcription factor Sip1 is accompanied by suppression of CaMKII and PI3K activity and leads to hyperexcitation of neuronal networks [41]. The mechanism by which CaMKII implements preconditioning can be pre- or postsynaptic in nature. In glutamatergic synapses, CaMKII phosphorylates many synaptic proteins and is involved in the synthesis and release of neurotransmitters. The lower level of extracellular glutamate in preconditioned cultures during ischemia is due to the action of CaMKII. In the post-synapse mechanism, CaMKII phosphorylates the NR2B NMDAR subunit, which is responsible for ligand binding [42]. All these effects of CaMKII are also realized during hypoxia and are an important component of hypoxic preconditioning [43,44]. In cerebral cortex neurons with a deletion of the transcription factor Sip1, hypoxic preconditioning is also impaired, and signs of epileptiform activity are recorded, which correlates with a decrease in CaMKII expression [45], which is probably also observed in the case of the deletion of Satb1.

It should be noted that the level of expression of the neurotrophin BDNF and its receptors (Trkb) is suppressed upon complete deletion of Satb1. At the same time, it is well known that BDNF, through branched signaling cascades involving Ras/MAPK/ERK (rat sarcoma protein/mitogen-activated protein kinase), PI3K/Akt, and PLC-γ1/PKC (phospholipase C-γ1/protein kinase C), contributes to the survival of neurons in conditions of oxidative stress. It has also been found that BDNF is involved in the induction of hypoxic preconditioning [20,46].

## 5. Conclusions

Deletion of the transcription factor Satb1 in neocortical glutamatergic neurons causes downregulation of the expression of key protein kinases. This, in turn, causes increased hyperexcitability of neocortical neurons and increased epileptiform activity in the magnesium-free and bicuculine models. Deletion of Satb1 is closely associated with hypoxic events and determines the increased sensitivity of Satb1-null neurons to a decrease in the oxygen concentration in the bloodstream. Disruption of hypoxic preconditioning in neurons of the cerebral cortex coincides with a change in the expression of the key protein kinases, CaMKII, PKC, PI3K, JNK, AKT, after deletion of Satb1. These changes in Ca^2+^ signaling and expression of signaling kinases can be involved in the induction of epilepsy, neurodegenerative diseases, and, in the case of severe stress on cells during hypoxia, can lead to the activation of apoptosis.

## Data Availability

The data presented in this study are available on request from the corresponding author.
